# Broad-host-range IncP-1 plasmids and their resistance potential

**DOI:** 10.3389/fmicb.2013.00044

**Published:** 2013-03-07

**Authors:** Magdalena Popowska, Agata Krawczyk-Balska

**Affiliations:** Department of Applied Microbiology, Institute of Microbiology, Faculty of Biology, University of WarsawWarsaw, Poland

**Keywords:** IncP plasmid, antibiotic resistance, heavy metals, xenobiotic, catabolism, horizontal gene transfer, bioremediation

## Abstract

The plasmids of the incompatibility (Inc) group IncP-1, also called IncP, as extrachromosomal genetic elements can transfer and replicate virtually in all Gram-negative bacteria. They are composed of backbone genes that encode a variety of essential functions and accessory genes that have implications for human health and environmental bioremediation. Broad-host-range IncP plasmids are known to spread genes between distinct phylogenetic groups of bacteria. These genes often code for resistances to a broad spectrum of antibiotics, heavy metals, and quaternary ammonium compounds used as disinfectants. The backbone of these plasmids carries modules that enable them to effectively replicate, move to a new host via conjugative transfer and to be stably maintained in bacterial cells. The adaptive, resistance, and virulence genes are mainly located on mobile genetic elements integrated between the functional plasmid backbone modules. Environmental studies have demonstrated the wide distribution of IncP-like replicons in manure, soils and wastewater treatment plants. They also are present in strains of pathogenic or opportunistic bacteria, which can be a cause for concern, because they may encode multiresistance. Their broad distribution suggests that IncP plasmids play a crucial role in bacterial adaptation by utilizing horizontal gene transfer. This review summarizes the variety of genetic information and physiological functions carried by IncP plasmids, which can contribute to the spread of antibiotic and heavy metal resistance while also mediating the process of bioremediation of pollutants. Due to the location of the resistance genes on plasmids with a broad-host-range and the presence of transposons carrying these genes it seems that the spread of these genes would be possible and quite hazardous in infection control. Future studies are required to determine the level of risk of the spread of resistance genes located on these plasmids.

## CHARACTERISTICS OF IncP-1 PLASMIDS

A characteristic feature of all plasmids, including IncP-1 plasmids, is that they belong to specific incompatibility (Inc) groups. Plasmids are classified into Inc groups based on their replication and partitioning systems: two plasmids belonging to the same Inc group are unable to coexist in a single bacterial cell (Shintani et al., 2010b).

Members of the *Enterobacteriaceae* family and the genus *Pseudomonas* carry plasmids belonging to more than 30 Inc groups. Plasmids from four of these groups (IncP, W, N, and Q) can transfer between and maintain themselves in both enteric bacteria and strains of *Pseudomonas *(Dröge et al., 2000). In particular, IncP plasmids are widely distributed in Gram-negative bacteria, e.g., in *Escherichia coli*, *Pseudomonas *spp., *Klebsiella aerogenes*, and *Sphingomonas* (Thomas, 2000; Shintani et al., 2010a). It should be added that IncP-1 in the *Pseudomonas* plasmid classification corresponds to IncP in the *E. coli* plasmid classification (Thomas and Haines, 2004).

The plasmids of the subgroup IncP-1 are of considerable interest to both molecular biologists and environmentalists due to their highly efficient conjugative transfer and ability to replicate in a broad range of hosts (Shintani et al., 2010a). These plasmids demonstrate great diversity – a phylogenetic study has shown the presence of 45 backbone genes on IncP-1 plasmids, but only 33 are shared by all the plasmids that have been completely sequenced to date.

A detailed phylogenetic analysis and study of the amino acid sequence of protein TrfA, which initiates plasmid replication, have allowed the classification of group IncP-1 into six subgroups: -α, -β, -γ, -ε, -δ (Bahl, 2009), and -ζ (Norberg et al., 2011) and also into an unnamed subgroup (Pachulec and van der Does, 2010). Unpublished data also indicate the existence of a η subgroup (Sen et al., 2012). IncP-1 plasmids have been identified in clinical and environmental, phylogenetically very distant, host bacterial species from around the world. Bacteria carrying these plasmids have been isolated from soil in areas contaminated by industry, pig manure, wastewater of industrial origin, river sediment, and fresh water (Bahl, 2009; Norberg et al., 2011; Sen et al., 2012). Comparative analysis of the nucleotide sequences of plasmids belonging to the IncP-1 subgroups α and β demonstrated the high similarity of their gene organization and also led to the identification of adaptive genes called “load genes.” The backbone sequences of these plasmids are responsible for the regulation of replication, stable maintenance in a bacterial cell and efficient conjugative transfer. In plasmids of the IncP-1β subgroup, the Tra1 and Tra2 regions of the backbone, that mediate conjugative transfer, are always separated by regions of clustered restriction sites, which may represent hotspots for the integration of various determinants. These regions can be a site for the insertion of mobile genetic elements (MGE) carrying catabolic genes, and antibiotic and heavy metal resistance determinants. The capacity for MGE integration is highly variable among IncP-1 plasmids. Plasmids pJP4, pSS60, and pSS50, belonging to the subgroup IncP-1β, contain one site for the integration of catabolic genes in their plasmid backbone, which is located between the *oriV *and *trfA* system replication sequences. The number of insertions in the backbone varies among IncP-1 plasmids (Trefault et al., 2004). pB4 is the only plasmid known to have three insertion sites: catabolic genes grouped into an operon are located near the *oriV* sequence, and antibiotic resistance determinants are located at both ends of region Tra2 (Adamczyk and Jagura-Burdzy, 2003).

There are currently only three known representatives of the subgroup IncP-1δ, namely plasmids pEST4011, pIJB1, and pAKD4. Plasmid pAKD4 is regarded as the prototype of this subgroup because it is the only one to possess the complete backbone sequence. Like other IncP-1 plasmids, those of subgroup δ carry specific genes that are located on transposons integrated within the plasmid backbone (Sen et al., 2010, 2012).

More than 50 plasmids belonging to the subgroup IncP-1ε have recently been identified and characterized. These plasmids confer resistance to a broad spectrum of antibiotics. This is due to the presence of a class 1 integron that carries the gene *sul1* responsible for sulphonamide resistance at its 5 ′ end, plus gene cassettes carrying several determinants of resistance to other antibiotics (Heuer et al., 2012). Plasmids belonging to subgroup IncP-1ε exhibit great diversity (Sen et al., 2011).

## ANTIBIOTIC RESISTANCE GENES PRESENT ON IncP PLASMIDS

The extensive use of antibiotics for the treatment of humans and animals, and to stimulate the growth of livestock, has led to the rapid emergence and spread of resistance determinants to a broad spectrum of antibiotic substances (Allen et al., 2010; Aminov, 2010; Popowska et al., 2010, 2012; Tello et al., 2012). These determinants are located mainly on MGE such as plasmids and conjugative transposons, which ensures their spread by horizontal gene transfer. Horizontal gene transfer involving the acquisition of foreign DNA by transduction, transformation, or conjugation, is quite common among bacteria and plasmids play a key role in this process (Aminov, 2011; Martinez, 2009; Stokes and Gillings, 2011). Among the plasmids conferring resistance to antibiotics, representatives of the incompatibility groups P, Q, N, and W have been identified. These are characterized by their wide host range, including pathogenic bacteria, and so pose a serious threat to human health (Dröge et al., 2000). Numerous studies have demonstrated that the prevalence of such plasmids in different environments is very high, e.g., in soil, surface waters, wastewaters, and natural fertilizers (Götz et al., 1996; Haines et al., 2006; Binh et al., 2008; Sen et al., 2011; Heuer et al., 2012).

There are four basic mechanisms of plasmid-encoded antibiotic resistance: (i) enzymatic hydrolysis of the antibiotic molecule, which results in inactivation of the compound, (ii) enzymatic modification of the antibiotic to prevent interaction with the cellular target, (iii) efflux, which removes the antibiotic from the interior of the cell via specific cell membrane pumps, and (iv) a reaction called “bypass,” which is the formation of an alternative metabolic pathway that allows bypassing of the blocked stage and modification of the antibiotic target (Alekshun and Levy, 2007). The genes encoding the proteins responsible for these mechanisms are found on plasmids present in both clinical and environmental bacterial isolates (Aminov and Mackie, 2007; Allen et al., 2010; Wright, 2010).

**Table 1** shows plasmids from IncP-1 group, well characterized in terms of phenotype and molecular mechanisms of observed resistance to antibiotics and heavy metals. Resistance plasmids classified within subgroup IncP-1α were first identified in hospital isolates of *Pseudomonas aeruginosa* and *K. aerogenes*. These were found to encode multi-drug resistance (MDR) against antibiotics including penicillin, kanamycin, and tetracycline (Adamczyk and Jagura-Burdzy, 2003). Using the exogenous plasmid isolation method Dröge et al. (2000) found resistance plasmids, including those belonging to the subgroup IncP-1α, in bacterial communities from activated sludge derived from wastewater treatment plant (WWTP). Twelve plasmids were identified carrying an antibiotic and heavy metal resistance determinant, and ten of these could be classified to group IncP. These studies confirmed the wide distribution of this Inc group in these environments.

**Table 1:** General features of IncP-1 plasmids carrying resistance genes.

Plasmid	Size (kb)	IncP-1 subgroup	Resistances	Resistance genes	Detected transposons	Reference/accession No.
pAKD1	58,246	IncP-1β	Sp, Sm, Su, Hg	*aadA, sul1, merE*	Tn*21*-like transposon	Sen et al. (2011)/JN106164.1
pB2/pB3^#^	60,732/56,167	IncP-1β	Aminoglycosides, β-lactam, Cm, Su, Tc, quaternary ammonium compounds	*aadA2, bla*_NPS_2_, cmlA1, sul1, tetA(C), tetR(C), qacE∆1	Tn-*tet, *Tn*402*	Heuer et al. (2004)/NC_006388.1
pB4	79,370	IncP-1β	β-lactam, tripartite multi-drug resistance (MDR) efflux system, Sm, Em, Chr	*bla_NPS_1_*, strAB, mexCD-oprJ, chr	Tn*5393c*, Tn*5719*	Dröge et al. (2000), Tauch et al. (2003)/NC_003430.1
pB5	64,696	IncP-1α	Sm, Tc, Km, Gm, Su, quaternary ammonium compounds	*aacA4, aacC1, tetA, tetR, aphA, sul1, qacE∆1*	nd	Dröge et al. (2000); Szczepanowski et al. (2011)/NC_019020.1
pB6	58	IncP-1β	Tc, Sm, Sp, Cm, Su	nd	nd	Dröge et al. (2000)
pB8	57,198	IncP-1β	Sm, Sp, β-lactam, Su, quaternary ammonium compounds	*aadA4, oxa2, sul1, qacE∆1, qacF*	Tn*5501, *“cryptic” Tn, Tn*402/ *Tn*5090*,Tn*QAC*/(Tn*3* family)*, *Tn*501*/Tn*21*	Schlüter et al. (2005)/NC_007502.1
pB10	64,508	IncP-1β	β-lactam, Su, Sm, Tc, quaternary ammonium compounds, Hg	*oxa2, sul1, strAB, tetA, qacE∆1, mer*	Tn*5393c*, Tn*1721, *Tn*501*	Schlüter et al. (2003)/NC_004840.1
pB11	66,911	IncP-1α	Tc, Ap, Km, Hg	*tetA, tetR, aphA, merE*	Tn*501,* Tn*5053*	Dröge et al. (2000), Szczepanowski et al. (2011)/CP002152.1
pB12	64,393	IncP-1β	Tc, Sm, Sp, Em, β-lactam / Su, quaternary ammonium compounds	*tetA, aacA4, oxa2, sul1, qacE∆1*	Tn*21*, Tn*402*	Dröge et al. (2000), Sen et al. (2012)/JX469826.1
pTB11	68,869	IncP-1α	Aminoglycosides, β-lactam, Tc	*aphA, aadA1, aacA4, oxa2, tetA, tetR*	Tn*402/ *(Tn*5090*), Tn*1721,*	Tennstedt et al. (2005)/NC_006352.1
pMCBF1	62,689	IncP-1ζ	Multi-drug efflux (MDE) outer membrane prot. NodT family, Hg	*oprN, merE*	Tn*5053*	Norberg et al. (2011)/AY950444.1
RP4/RK2	60,099	IncP-1α	Tc, Km, Ap	*tetA, aph*	Tn*4371*, Tn*1*	Pansegrau et al. (1994)/L27758.1
pTH10	70	IncP-1α	Tc, Km, Ap	nd	nd	Harayama et al. (1980)
R751	53,423	IncP-1β	Tp	*dhfrIIc*	Tn*402/ *Tn*5090*, Tn*501*	Thorsted et al. (1998)
pKJK5	54,383	IncP-1ε	Tc, Tp, aminoglycosides, Su, Sp, quaternary ammonium compounds	*tetA, tetR, dfrA1, aadA11b, sul1, qacE∆1*	Tn*402*	Bahl et al. (2007)/NC_008272.1
pG527	80,762	IncP-1α	Aminoglycosides, Km, Sm, β-lactam, Tc	*aadA1, aphA, sph, *bla _*TEM-67*_, tetA, tetR	Tn*3*, Tn*7*, Tn*1721*	Sen et al. (2012)/JX469830.1
pSP21	72,683	IncP-1α	Tc, Km, aminoglycosides, β-lactam	*tetA, tetR, aph, aadA1, aacA4, oxa2*	Tn*402*	Pansegrau et al. (1994),Szczepanowski et al. (2011)/NC_019021.1
pBS228	89,147	IncP-1α	Aminoglycosides, Sp, Tp, Tc, β-lactam, Hg	*aadA, dhfr, tetA,*bla*_TEM-67_*, aph, merE	Tn*1013*, Tn*5718*, Tn*1*, Tn*7*	Haines et al. (2007)/NC_008357.1
BRA100	56,265	IncP-1β	Aminoglycosides, Su, quaternary ammonium compounds, Hg	*aacA4, strAB, sul1, qacE∆1, merE*	Tn*6305*/(Tn*3* family)	Unpublished/CP003505
pWEC911	74,056	IncP-1	Tc,* β*-lactam, Km, Hg	*tetA, *bla_*TEM-67*_, aphA, merE	nd	Sen et al. (2012)/JX469833.1
pKSP212	54,342	IncP-1	Aminoglycosides, Su*, Hg*	*aac(6)-Ib, sul1, merE*	Tn*3*	Sen et al. (2012)/JX469831.1
pBRSB222	36,880	IncP-1	Aminoglycosides, β-lactam, Su, quaternary ammonium compounds	*aadA5, oxa2, sul1, qacE*∆*1*	nd	Sen et al. (2012)JX469825.1
pKS208	50,604	IncP-1	Aminoglycosides, Km, β-lactam	*aac(6)-Ib, aphA1, pEC-IMPQ_139*	Tn*1525*, Tn*5053*/Tn*402*	Sen et al. (2012)/JQ432564.1

*Ap, ampicillin; Cb, carbenicillin; Cm, chloramphenicol; Gm, gentamicin; Km, kanamycin; Nx, nalidixic acid; Sm, streptomycin; Sp, spectinomycin; Su, sulfanilamide; Tc, tetracycline; Tp, trimethoprim; Tm, tobramycin; Hg, inorganic mercury; nd, not determined.*

# *Plasmids pB2 and pB3 differ only by a duplication of a tetA(C)-tetR-tnpAIS26 fragment in pB2*.

To date, the complete nucleotide sequences of six resistance plasmids belonging to subgroup IncP-1α have been determined. These plasmids, RP4/RK2, pTB11, pBS228, pB5, pB11, and pSP21, show a high degree of similarity with respect to elements of their plasmid backbone, while they are distinguished by the nature of the genes located within their adaptive modules (e.g., ISTB11, ISSP21, Tn*402* derivatives, Tn*1*, Tn*7*). Plasmid pB5 contains an integron of class 1 carrying a resistance cassette with *aac*C1 and *aacA4 *genes, which encode enzymes that provide resistance to kanamycin, gentamicin, and tobramycin (Szczepanowski et al., 2011). The IncP-1α plasmids contain different variants of the integron *aadA* gene cassette (*aadA1*, *aadA2*, *aadA4*, and *aadA5*), which encode streptomycin/spectinomycin-300-*O*-adenylyltransferases conferring resistance to the aminoglycosides streptomycin and spectinomycin (Tennstedt et al., 2003, 2005). The *aadA2* gene cassette is widely distributed, having been identified in the integrons of many bacteria belonging to at least 11 genera. Numerous studies have demonstrated that different environmental and pathogenic bacteria share a common pool of integron-specific resistance gene cassettes (Tennstedt et al., 2003, 2005).

In the case of plasmids pTB11 and pSP21, a class 1 integron carrying genes that determine bacterial resistance to aminoglycosides is located within transposon Tn*402*. In addition, these plasmids possess a region containing genes for tetracycline resistance (*tetA* and *tetR*; Tennstedt et al., 2005; Szczepanowski et al., 2011).

The IncP-1α plasmid pTB11 carries the *aphA-orf3* module, encoding aminoglycoside-30-phosphotransferase responsible for aminoglycoside resistance, that shares the highest degree of similarity with the corresponding gene of plasmid RP4, originating from a clinical isolate. Another aminoglycoside resistance gene cassette located on pTB11 and pSp32 encodes an aminoglycoside-60-*N*-acetyltransferase mediating resistance to different aminoglycosides such as tobramycin, amikacin, and gentamicin. Two other aminoglycoside resistance gene cassettes, *aadB* and *aacA29b*, which respectively, code for an aminoglycoside-200-*O*-adenylyltransferase and an aminoglycoside-60-*N*-acetyltransferase, have also been identified on IncP-1α plasmids. The *aadB* gene was found on a plasmid from a clinical isolate of *Acinetobacter baumannii*, while an *aacA29b* gene cassette, present on a plasmid, represents a new variant of *aacA29a* previously found on In59 in a clinical *P. aeruginosa* isolate (Siarkou et al., 2009).

Resistance plasmids are also represented in subgroup IncP-1β. These include the fully sequenced plasmids pB2/pB3, PB4, PB8, and PB10. These plasmids carry a class 1 integron or, in the case of PB4, a fragment of Tn*402*. Besides *sul1* that confers resistance to sulfonamides, these plasmids may also contain genes for resistance to other antibiotics and chemotherapeutics, e.g., *oxa* (PB8, PB10, and pB11) and *bla* (PB4 and PB3), encoding β-lactamase enzymes responsible for resistance to β-lactam antibiotics. Parts of the *oxa1* gene cassette in plasmid pSp33 and *blaNPS1* on the Tn*402* remnant in this plasmid are nearly identical to corresponding regions identified in different Korean clinical isolates of *Salmonella enterica* ssp. *enterica* (Lee et al., 2003, 2004) and *P. aeruginosa* (Jacoby and Matthew, 1979; Pai and Jacoby, 2001), respectively. However, some *bla* genes identified in this subgroup of plasmids have novel sequences. All of the aforementioned IncP-1β plasmids also carry genes encoding resistance to streptomycin, a member of the aminoglycoside antibiotic family. Plasmids PB4 and PB10 contain transposon Tn*5393c*, which includes the genes *strA* and *strB,* encoding aminoglycoside-3*′′*-*O*-phosphotransferase and aminoglycoside-6-*O*-phosphotransferase, respectively, that modify the streptomycin molecule, thus causing its inactivation. Identical or almost identical copies of Tn*5393* were recently identified in different human, fish, and plant pathogens. Plasmid pB10 also possesses the tetracycline resistance transposon Tn*1721*, that was first described in *E. coli* (Allmeier et al., 1992). A very similar transposon has been found in conjugative plasmids from the fish pathogen *Aeromonas salmonicida* (Sørum et al., 2003), the human pathogen *Salmonella enterica* ssp. *enterica* (Chiu et al., 2005) and a clinical isolate of *E. coli* (Boyd et al., 2004). With the use of plasmid pB10, carrying amoxicillin, streptomycin, sulfonamide, and tetracycline resistance genes, is has been shown that this broad-host-range IncP-1 plasmid can be transferred to foodborne pathogens (*Salmonella* spp. and *E. coli* O157:H7) under laboratory conditions (Van Meervenne et al., 2012). These results show that the antibiotic resistance genes encoded on plasmid pB10 can be expressed in the new hosts (pathogenic organisms) making these pathogens resistant to multiple antibiotics. These studies have shown that there is a serious risk to human health in cases of such transfer. The plasmid PB3 carries the gene *clmA1 *conferring resistance to chloramphenicol. The product of the *clmA1* gene is a protein that acts as an efflux pump, which removes the drug from the bacterial cell (Schlüter et al., 2007). The genetic modules on these IncP-1β plasmids consist mainly of MGE that carry diverse determinants conferring resistance to different aminoglycosides, β-lactams, macrolides, sulphonamides, tetracycline, trimethoprim, disinfectants, and mercury ions. The greatest MGE diversity has been observed in plasmids of this subgroup isolated from bacteria living in WWTP (Szczepanowski et al., 2004). WWTP bacteria represent a reservoir for β-lactamase genes that have yet to be isolated from clinical strains. Mobile integron gene cassettes seem to function in the dissemination of β-lactamase genes in sewage habitats.

An important role in the spread of resistance genes in agricultural systems is played by representatives of subgroup IncP-1ε. The study of Heuer et al. (2012) showed that fifty plasmids belonging to this subgroup, isolated from samples of soil and manure, contain integrons of class 1 with gene cassettes plus the gene* sul1 *(sulphonamide resistance). The nucleotide sequences of four of these plasmids have been determined (pKS77, pHH3414, pHH128, and pHH3408) and they differ mainly in the organization of the elements in transposon Tn*402*. These elements are a class 1 integron IS1326 and, in the case of plasmids pHH3414, pKS77, and pHH128, transposon Tn*1721* carrying the tetracycline resistance genes *tetA* and *tetR*. The resistance integrons contain the gene cassettes *aadA,* (pHH3414), *aadB* (pKS77), *aadA1b*, *dfrA1b*, and two copies of *catB* (pHH128; Heuer et al., 2012). The different *aad* gene variants encode adenyltransferases that modify the streptomycin and spectinomycin molecules, resulting in their inactivation. A similar mechanism is employed by the chloramphenicol acetyltransferase encoded by the *cat* genes to confer resistance to chloramphenicol. The *dfr* gene permits bacterial cells to survive in an environment with high concentrations of trimethoprim (Schlüter et al., 2007).

Interestingly, many studies have demonstrated a close relationship between the occurrence of MDR and resistance/tolerance to heavy metal ions (Lazar et al., 2002). Bacterial strains isolated from agricultural soil receiving wastewater can exhibit high levels of both metal and antibiotic resistance. Shafiani and Malik (2003) isolated 64 bacteria belonging to the genera *Pseudomonas*, *Azotobacter*, and *Rhizobium* from wastewater-irrigated soil. All the *Pseudomonas* isolates were resistant to heavy metals and cloxacillin, and more than half were also resistant to methicillin. Among these strains were MDR isolates. Ansari et al. (2008) found that 40 strains isolated from agricultural soils irrigated with wastewater showed resistance to most of the tested heavy metals (Ni, Cu, Zn, Pb). Moreover, around 75% of these strains were resistant to tetracycline, 57.5% to doxycycline, and 50% to ampicillin and nalidixic acid. The plasmids isolated from these resistant strains all belonged to the IncP group. Out of 12 pAKD plasmids (IncP-1 group) isolated from Norwegian soils only one, pAKD1, encoded antibiotic resistance in addition to mercury resistance. This plasmid carries a MDR Tn*21*-like transposon with a class 1 integron (Sen et al., 2011). These data suggest heavy metals treatment might have co-selected for MDR genes. This is consistent with previous and present observations that pollution with heavy metals can cause a rise in the abundance of drug resistance genes (Baker-Austin et al., 2006; Seiler and Berendonk, 2012).

## CONCLUSION

The increasing use of antibiotics in human and veterinary medicine and agricultural production systems has caused the increasing development of high levels of and novel antibiotic resistances and the rapid global spread of antibiotic resistance genes (Aminov, 2009, 2010; Martinez, 2009; Schlüter et al., 2007; Sen et al., 2011). A consequence of the horizontal transfer of the genes located on MGE, especially on Broad-host-range plasmids is the rising detection and levels of resistant bacteria, particularly of MDR bacteria, both in clinical and natural environments (Dröge et al., 2000; Aminov and Mackie, 2007).

IncP-1 plasmids are characterized by their wide distribution in the environment and ability to efficiently replicate in the cells of diverse hosts. In addition, the presence of transposons carrying both antibiotic and heavy metal resistance genes on these plasmids permits the host bacteria to survive and multiply in environments contaminated with these compounds. The broad-host-range IncP-1 plasmids carry determinants for resistance to at least one heavy metal (Ni, Cd, Co, Cu, Hg, Pb, Zn), and antibiotics of different groups, i.e., tetracyclines, quinolones, aminoglycosides, sulfonamides, β-lactams, and chemotherapeutics. This fact is not surprising in light of recent data showing that heavy metals present in the environment might have co-selected for MDR genes (Seiler and Berendonk, 2012). The two main mechanisms of antibiotic resistance encoded on IncP-1 plasmids are mediated by efflux pumps and enzymatic modification or hydrolysis of the antibiotic molecule. IncP-1 plasmid-containing bacteria are also key players in the horizontal transfer of antibiotic resistance in agricultural systems. It has been suggested that the spread of multi-resistant bacteria in soil, water, and WWTPs is mainly due to their possession of IncP-1 plasmids (Schlüter et al., 2003, 2005; Bahl et al., 2007; Ansari et al., 2008; Norberg et al., 2011; Sen et al., 2011).

Based on their DNA sequences, it can be concluded that the vast majority of these resistance genes are located on MGE such as transposons and/or integrons (Tn*402*-like transposon with a class 1 integron), embedded within these transposons. In addition, the presence of different combinations of MGE and integrons in IncP plasmids as well as of characteristic hot spots for the integration of accessory elements creates unlimited possibilities for rearrangements of the genetic material of these plasmids. It therefore appears that the spread of resistance genes would be possible and quite hazardous in infection control. Literature data show that many representatives of this group of plasmids carrying resistance genes have been identified in pathogenic bacteria of the genus *Escherichia*, *Salmonella*, *Shigella*, *Klebsiella*,* Aeromonas*, and *Pseudomonas*. Of course, in order to determine the level of risk of the spread of resistance genes located on these plasmids extensive experimental studies are required. However, the demonstrated ability to transfer multiresistance pB10 plasmid, isolated from a WWTP, to the foodborne pathogens *Salmonella* spp. or *E. coli* O157: H7 with expression of most of the transferred resistance genes in these strains poses a real threat to the life and health of people (Van Meervenne et al., 2012).

Detailed analysis of IncP-1 plasmid genomes can shed light on the adaptive mechanisms underlying bacterial evolution and this information may be used to develop effective methods of bioremediation of contaminated land with xenobiotic and antibiotics and heavy metals. Intensive research on the biodegradation of compounds that are a serious threat to the environment has led to the isolation and characterization of bacterial strains that can use these substances as a source of carbon and energy. In many cases, the adaptation of these microorganisms is due to plasmids (mainly of Inc group P) that carry genes encoding enzymes involved in the degradation of xenobiotics. It is known, that the degradative plasmids (IncP-1, P-7, and P-9) contain catabolic genes, often arranged as operons, encoding enzymes required for the degradation and utilization of many toxic compounds including naphthalene, toluene, chlorobenzene, p-toluenesulfonate, 2,4-D, haloacetate, and atrazine (Williams, 2004; Shintani et al., 2010a,b). The majority of bacterial genes encoding enzymes of catabolic pathways are located on MGE such as plasmids, insertion sequences (e.g., as a form of composite transposons), transposons, integrons, genomic islands, and phage genomes (Top et al., 2002; Top and Springael, 2003; Nojiri et al., 2004; Springael and Top, 2004).

Theoretically, genetic modules present in this type of element (both in catabolic and resistance plasmids) may be used in the construction of genetically modified strains that are optimized for use in bioremediation and biotechnology (Vidali, 2001; Urgun-Demirtas et al., 2006; Singh et al., 2011). This is of great importance due to the fact that these antimicrobial agents are powerful inhibitors of xenobiotic biodegradation activities, mainly due to their bacteriostatic or bactericidal activities. However, before the broader implementation of such microorganisms, further research is required to thoroughly characterize them in order to limit the spread of antibiotic resistance genes.

## Conflict of Interest Statement

The authors declare that the research was conducted in the absence of any commercial or financial relationships that could be construed as a potential conflict of interest.
